# A report on the genital myiasis by *Wohlfahrtia magnifica* in camel herds in southwest of Iran

**Published:** 2014

**Authors:** Khodadad Pirali Kheirabadi, Amir Dehghani Samani, Hossein Rajabi Vardanjani

**Affiliations:** 1 *Department of Pathobiology, Faculty of Veterinary Medicine, Shahrekord University, Shahrekord, Iran; *; 2 *Department of Clinical Sciences, Faculty of Veterinary Medicine, Shahrekord University, Shahrekord, Iran; *; 3 *Department of Basic Sciences, Faculty of Pharmacy, Student Research Committee of Ahvaz Jundishapur University of Medical Sciences, Ahvaz, Iran.*

**Keywords:** Camel, Iran, Larvae, Myiasis, *Wohlfahrtia magnifica*

## Abstract

Myiasis is a disease of vertebrate animals caused by different fly larvae. *Wohlfahrtia magnifica* is responsible for serious losses in animal husbandry in Eurasia. Larvae of *W. magnifica* parasitize several warm-blooded vertebrates and are responsible for a severe traumatic myiasis of mucosal membranes or wounds. This myiasis has been reported in many European areas, but for the first time was reported from Iran. Larvae infestation was observed in five camels out of 35 (14.28%) around the perinea and vaginal region of camels in a herd in southwest of Iran. The larvae samples were collected and transferred to the laboratory of parasitology for identification. This is the first report of infestation of a camel herd (*camelus dromedarius*) in Iran. The treatment was performed and prevented the loss from camels and improved their physical condition. Although the myiasis is not a lethal disorder, however knowledge of the disease is necessary from preventive, diagnostic and curative standpoint.

## Introduction

Myiasis is a rare disease that broadly includes interaction between flies and warm blooded hosts. It usually involves infestation by larval or pupal stages of the flies in the tissues of human and other vertebrate animals.^1^

Infestation with fly larvae may occur when flies deposit eggs or first-stage larvae on the body or its apertures. The clinical aspects of myiasis vary with the regions affected, with the species of fly involved and with the number of maggots present. Myiasis is more prevalent in tropical regions. Myiasis with different causative agents is common in domestic and wild mammals all over the world.^2^

The most important agent of myiasis in the genus *Wohlfahrtia *is *W. magnifica*, an obligatory parasite of warm- blooded vertebrates in southeastern Europe, southern and Asiatic Russia, the near east, north Africa and Eurasia.^3^

Larvae stage of *W. magnifica* parasitize several warm-blooded vertebrates and are responsible for a severe traumatic myiasis of mucosal membranes or wounds. This myiasis has been reported in many European areas, from west to far eastern countries to Morocco and northern Asia.^4^^,^^5^

In Europe, larvae of flesh fly *Wohlfahrtia* spp, affect mainly sheep, with up to 90.00% of flocks infested and prevalence in animals ranging from 4.70% to 38.90%. This myiasis causes severe economic losses to farmers due to the failure of animal conditions, reproduction problems, lameness, blindness and even death of infested sheep.^6^

Wohlfahrtiosis has also been described in goats,^7^ horses,^8^ dogs,^9^ geese,^10^ pigs,^11^ camels^10^ and in a leopard.^11^ Additionally, larvae of *W. magnifica* have been reported as an agent of myiasis even in humans in Mediterranean countries.^12^^,^^13^

In a survey on screwworm fly distribution in ruminants in south-western Europe, *W. magnifica* adult specimens were captured using bait traps in a south-western Italian region.^14^^,^^15^

The present report describes presence of camel infestation with maggots of *W. magnifica *in southwest of Iran.

## Materials and Methods

In Jun 2010, infestation by maggots in a camel herd containing 35 camels was reported from a region in Iran. Five 2-3 years old camels (three alive and two dead) were infested by maggots. This region (latitude, 32° 27' 06" N and longitude, 50° 54' 38" E) is located in southwest of Iran with altitude over 2000 meters above sea level.

Clinical examinations were carried out on the herd which suffered from extreme cachexia. The other vital signs of herd were normal. The fly larvae were observed around the perinea and vagina. A total numbers of 50 larvae were removed from injuries on the examined camels using sterile forceps and kept in ethanol 70%. The maggots were subjected to identification acoording to the key identification by Belding^16^ using stereo microscope (Olympus CX41, Tokyo, Japan) at 40×. Necropsy was also performed on the infested camels.

## Results

**Fig. 1:**
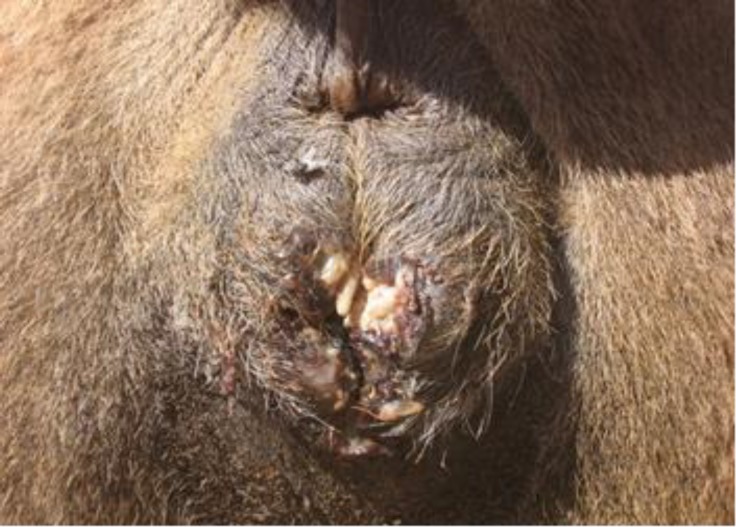
Myiasis around the perinea and vaginal region of infested camels

**Fig. 2:**
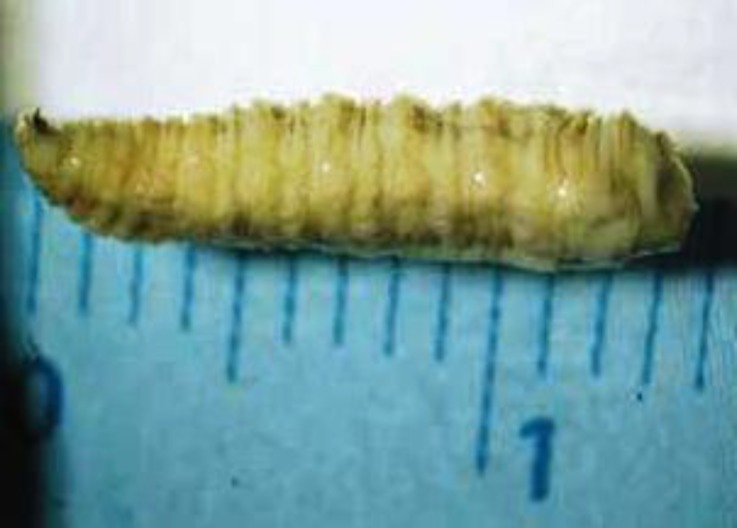
Size and color of maggots. Maggots were cylindrical with the flattened ventral surfaces

Maggots were observed in five out of 35 (14.28%) camels around the perineal and vaginal region. There were 33 to 50 larvae in perinea area and vaginal tissue. Signs of bleeding and septic wounds caused by myiasis were not observed (Fig. 1). Myiasis in other parts of the infested camels’ body was not found. There were no important necropsy findings. Three camels had severe myiasis with a lot of maggots around perinea area and vaginal tissue. Macroscopically, examined maggots were belonging to the third stage larva of *W. magnifica*, grayish in color, with 12 to 18 mm in length and 3 to 4 mm in width (Fig. 2). The maggots were cylindrical in shape with flattened ventral surface with cephalopharyngeal sclerite. 

**Fig. 3:**
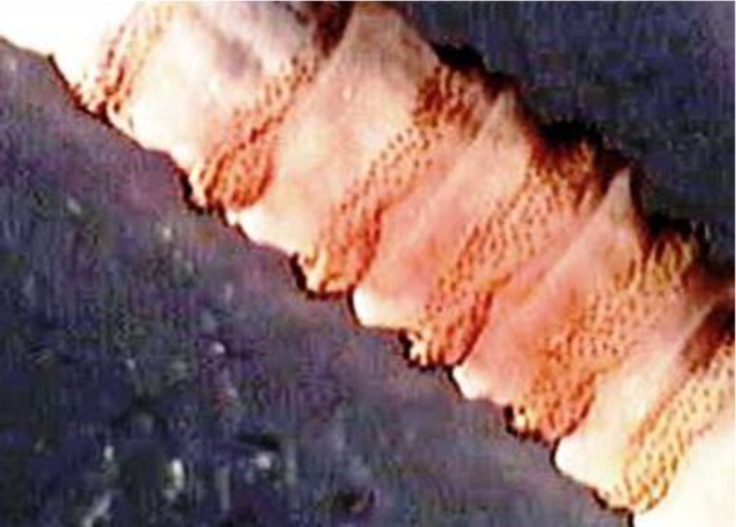
Maggots were cylindrical, which spines located between each segments of body.

**Fig. 4:**
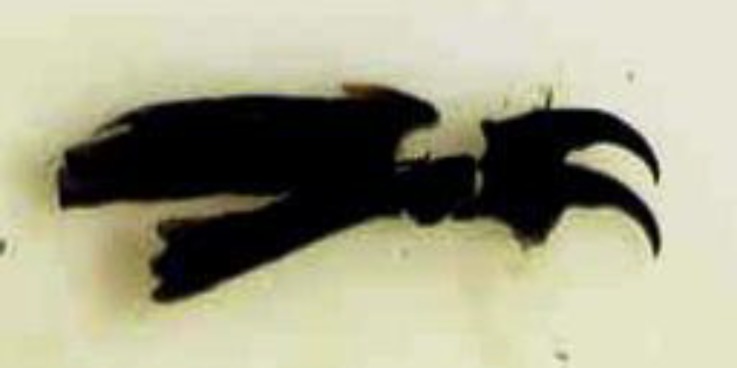
Special cephalopharyngeal skeleton of maggots

**Fig. 5.**
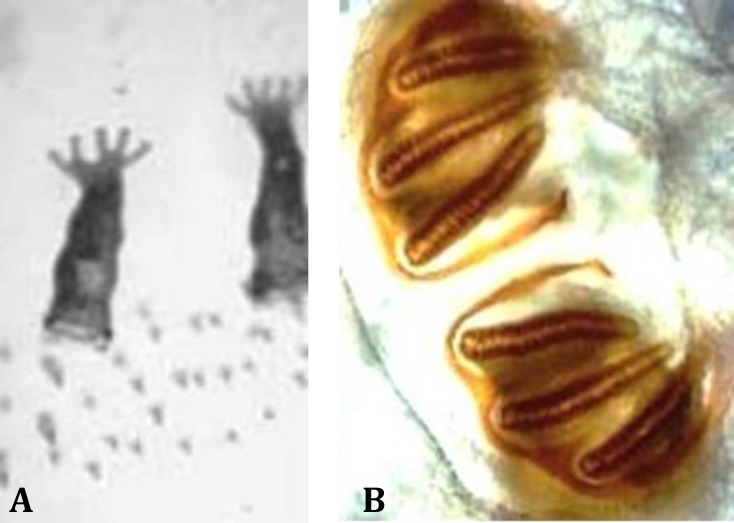
**A. **Anterior spiracles of maggots which have five branches. **B.** Posterior peritremes are elongated in the dorsal surface of the end somatic segment. The peritremes have three variably shaped peritremal splits, the posterior spiracles were located near each other and each plate was formed of widely opened peritreme

The spines were located in each body segment (Figs. 3 and 4). The anterior spiracles had five branches (Fig. 5A). The posterior peritremes were elongated on the dorsal surface of the somatic segment. There was three shapes of peritreme splits in which the posterior spiracles were nearly located to each one. The plate was formed with wide openings on the peritreme (Fig. 5B).

Myiasis treatment was performed with removing the larvae, washing and cleaning up the injuries. Oxytetracycline (10 mg kg^-1^, IM; Erfan Co., Tehran, Iran) and ivermectin (0.2 mg kg^-1^, SC; Razak Co., Tehran, Iran) were also injected.

## Discussion

This study showed that myiasis by *W. magnifica* can be very important disease in herds and can have many effects on camels, and may result in death.

Myiasis affects animals’ health and causes severe economic losses such as abortion, reduction of milk production and losses in terms of weight gain, fertility, and hide quality to the livestock industry in both of developing and developed countries. Some myiasis can be life-threatening also for humans, mainly in developing areas. While the diagnosis of the vast majority of the animal ectoparasitosis is achieved by the visual detection of parasites on the animal’s body (e.g. flea and tick infestation, pediculosis) or in biological samples (e.g. mange mites detectable in scabs), this is not the case for the majority of oestrids causing internal myiasis. In fact the diagnosis of myiases depends on the anatomical location of larvae in the host body and is usually carried out by the parasitological examination (e.g. palpation of animals’ back for warble fly hypodermosis in domestic and wild ruminants) or by a post-mortem examination of internal organs (e.g. horse gasterophilosis, sheep oestrosis).^17^

Some larvae are deposited near the wounds or body openings of man and animals like sheep, goat, cattle, horse, donkey, pig, dog, camel and goose. The larvae of *W. magnifica *feed and mature in 5 to 7 days and then leave the wound for pupation.^18^ *Wohlfahrtia nuba *also infests wounds of livestock in north Africa and Middle East, but it probably feeds only on dead or diseased tissues rather than on living tissues. *Wohlfahrtia vigil *and *W. meigeni *(opaca) are north American species whose larvae tend to penetrate the hosts skin individually producing furuncles like Cordylobia. Wohlfahrtia meigeni can be a serious pest of mink and fox in fur farms in north America.^19^

Etiologically, myiasis are classified into three groups according to characristics of the infested tissues of which *Wohlfahrtia *is as obligatory parasite.^20^

*Wohlfahrtia magnifica *larvae infest the ear, eye and nose with damaging tissues. The infestation frequently produces furunculous or boil-like lesion in subcutaneous area, however, it may occur on the skin wounds and body cavities.^21^

In conclusion, myiasis could not be detected by common examinations usually, so clinicians should be more alert to the possibility of presence of myiasis in every part of animal’s body. Also, animals should be monitored for myiasis and in infestation with maggots treatment should be performed for the infested animals and herds.
